# Nutrient-Aware Personalized Meal Recommendation Using Structured Food Knowledge and Constraint Verification

**DOI:** 10.3390/foods15101647

**Published:** 2026-05-09

**Authors:** Yu Fu, Linyue Cai, Ruoyu Wu, Yongqi Kang, Yong Zhao

**Affiliations:** College of Computer Science, Sichuan University, Chengdu 610207, China; fuyu05@stu.scu.edu.cn (Y.F.); linyuecai811@gmail.com (L.C.); 2023141520216@stu.scu.edu.cn (R.W.); 2023141520239@stu.scu.edu.cn (Y.K.)

**Keywords:** personalized nutrition, meal recommendation, food knowledge graph, dietary constraints, lightweight language models, intent refinement

## Abstract

Along with the enhancement of people’s public health consciousness and the requirement for individual diet arrangement getting more urgent, the meal recommendation method, which is based on artificial intelligence, has hence become an active research domain in the field of intelligent health. One system that makes practical recommendations must deal with the user’s unclear queries, while at the same time, it must satisfy strict nutrient demands. A great number of existing methods at present either do not take into account verifiable food composition data, or they handle implicit dietary restrictions in a not good way. For solving these problems, we put forward CARE (Constraint-Aware Recipe Engine). Beginning from a mixed Retrieval-Augmented Generation (RAG) basic model (CARE v1.0), we have developed CARE v2.0, which is a suggestion engine that unites intention polish, knowledge graph enlargement, and rule-based checking in a unified working line. Instead of depending on huge black-box models, our framework utilizes an effective language model that possesses 1.5 B parameters. User inquiry content are undergone parsing to become structured nutrition targets; a food knowledge graph links abstract health notions to specific cooking materials; and the obtained candidate results are filtered in accordance with strict diet restrictions, with optional checking carried out by an automatic agent-based reviewer. Under a zero-shot cold-start situation, the system attains a semantic recall@5 of 0.825 on 400 k recipes coming from Recipe1M+ and a newly created fuzzy-query benchmark (CAREBench-150), and it thus has a better performance than dense retrieval baselines (0.550) as well as direct zero-shot prompting. The constraint satisfaction rate is located at 85.0% in fast mode, and it rises to 98.5% when the verification module is in the working state; therefore, it supports the safety of recommendations. These findings indicate that structured food knowledge, which matches a compact algorithmic framework, can therefore connect unclear user intentions and accurate nutrition requirements effectively.

## 1. Introduction

Choice of diet has a strong influence on health, yet changing general nutrition guidelines into concrete, healthy dishes is still a hard thing for many people. When users look for recipes, they are inclined to put forward their requirements with ambiguous expressions like “something light but filling,” “a quick family dinner,” or “a dish that supports immune health.” These question requests carry many different dimensions together: nutritional goals (low calorie, high satiety), practical constraints (cooking time, servings), and sensory preferences (texture, flavor). Constructing systems that can steadily explain this uncertainty and give nutritionally healthy suggestions is a key difficulty at the crossing point of food science and artificial intelligence [1]. Despite growing interest in this area, no existing open-source framework simultaneously handles fuzzy intent interpretation, verifiable nutritional grounding, and strict constraint enforcement within a single lightweight pipeline.

The current methods for giving food suggestions, in a big way, can be divided into three types, each of which has obvious shortcomings. Knowledge Graph (KG) approaches [2,3] make use of structured culinary connections to promote explainability; however, they usually require well-formed inputs like precise ingredient lists and process open-ended natural language in a bad way. Generative systems constructed upon Large Language Models (LLMs) [4,5] have conversational smoothness but often “hallucinate” cooking methods that break strict dietary restrictions because they have no foundation in checkable food component data. Retrieval-Augmented Generation (RAG) [5,6] in part solves this gap through looking up external documents, yet the majority of RAG-based food systems [7] take retrieval as simple text matching, and neglect the abundant relational data which can be obtained in food knowledge bases. Frameworks such as DietQA [8] manifest the worth of combining KGs with LLMs for multi-diet question answering, but these frameworks are not designed for open-ended, fuzzy recommendation tasks.

The problem of recipe recommendation based on fuzzy intention has several challenges that existing models have not solved. The first one is the ambiguity of intention, which produces a semantic gap [9]: a query such as “light but filling” needs fine-grained comprehension that is beyond keyword matching, hence the system must coordinate low-calorie restrictions with high-satiety food materials. The second difficult problem is that carrying out nutritional restrictions needs provable basic support, hence this means suggested plans must match real food ingredient information instead of experiential text making. The third item is explainability: in applications related to health, users need to understand why a specific recipe has been recommended, especially when strict dietary limitations, such as allergies, are present.

To answer these challenges, we put forward CARE. We carry out the division of system development into two phases in order to clearly manifest the value of each architectural innovation. CARE version 1.0 acts as the basic foundation baseline through using one standard hybrid RAG framework that has no graph-based knowledge or adaptive weight distribution. To address the semantic gaps in v1.0, we have introduced CARE v2.0, which is a recommendation engine that combines structured food knowledge with constraint-driven retrieval. CARE version 2.0 adopts a modular construction which takes parameter efficiency as the core. Instead of putting faith in large-scale, non-transparent LLMs, for example, GPT-4 or Llama-3, it utilizes a light-weight 1.5 B-parameter model (Qwen2.5-1.5B-Instruct).

The core creative points of v2.0 are: (i) one query-to-constraint translation module which changes blurry user inputs into clear nourishment goals; (ii) one food knowledge graph that connects abstract healthy concepts to actual materials for cooking; (iii) one adaptive retrieval mechanism which carries out the balance between keyword accuracy and semantic recall; and (iv) one optional step of automatic verification which is for requests that are critical to safety. These constituent parts make up a four-step flow process that we name Refine, Retrieve, Rerank, and Reason (4R).

The present investigation is directed by three research questions (RQs):

RQ1 (Semantic Reasoning): Can structured food knowledge graphs, which are combined with adaptive retrieval, bridge the semantic gap for vague dietary intents in a more effective way than traditional sparse and dense retrieval?

RQ2 (Verifiable Safety): When we augment a parameter-efficient 1.5 B language model with rule-based hard filtering and one agent-based verification module, can it meet strict dietary constraint satisfaction, including the avoidance of allergens?

RQ3 (User Satisfaction): Whether the combination of constraint-aware retrieval and verifiable reasoning promotes the entirety of nutrition appropriateness and satisfaction that users feel in the actual recommendation situations?

Our main contributions are as follows:

- A Parameter-Efficient 4R Architecture. We put forward CARE v2.0, it makes probabilistic intent parsing separate from deterministic safety filtering by means of a Refine–Retrieve–Rerank–Reason working flow. This scheme enables a compact 1.5 B-parameter model to undertake complicated recommendation work in the absence of large-scale calculation resources.
- Bridging the Semantic Gap with GraphRAG. We put forward an adaptive retrieval mechanism which dynamically keeps a balance between semantic dense embeddings and structured Food Knowledge Graph (Recipe1M+ and USDA) traversal, hence improving retrieval accuracy for fuzzy, implicit health intentions.
- Verifiable Dietary Safety via Agentic Critic.. We have designed a dual safety mechanism that combines rigid rule-based hard filtering and an autonomous Agentic Critic. This thereby promotes the constraint satisfaction rate to 98.5% for safety-critical queries, therefore providing a dependable protection measure for the management of diet.
- An Open-Source Benchmark. We have constructed and released CAREBench-150, which is a benchmark that possesses human-annotated relevance labels that allow standardized assessment of fuzzy dietary intents.

The outcomes of the experiment prove that CARE v2.0 has a good effect. Under fast working mode, it obtains the same result as BM25 in exact-match tasks, and at the same time it performs better than dense retrieval in fuzzy queries (SR@5 is 0.825 compared with 0.550). When the agentic verification module is opened, the constraint satisfaction can reach a relatively high safety level, which therefore shows that strong reasoning accuracy and dietary safety do not certainly need large-scale models with expensive computation costs.

## 2. Related Work

### 2.1. Recipe Recommendation Paradigms

Traditional recipe recommendation systems heavily depend on collaborative filtering (CF) [10] or content-based filtering (CBF) [11], these two kinds all suppose that user preferences are static and are captured in an explicit way in interaction logs. Newest summarizations on food computing [7] give wide classification systems but frequently put traditional heuristic filtering (e.g., elementary CF or boolean tag matching) together with modern neuro-symbolic architectures (e.g., LLMs which are augmented by KGs) under the umbrella term “hybrid systems.” This dividing method has problems because it hides a basic change in technology: unlike traditional ways, which passively get recipes according to fixed past related connections, advanced agentic frameworks do active thinking on clear, changing nutritional constraints. In the academic literature, there is still an explicit request for distinguishing systems that possess the capability to perform verifiable, safety-critical dietary reasoning from conventional passive recommendation algorithms. For solving the problem of data sparsity, the methods which are based on KG utilize the structured relationships of the cooking domain. FoodKG [2] and the works which came after it [3,12] utilize KGs for promoting explainability and reasoning, and heterogeneous graph models have been constructed to utilize relation information among users, recipes, ingredients [12]. These methods, nevertheless, normally demand structured input data and have difficulties when dealing with open-type natural language queries.

### 2.2. Neuro-Symbolic and RAG Approaches in Food AI

The appearance of LLMs has made research move in the direction of generative recommendation. Systems such as ChatDiet [13] have smooth conversation ability but often fabricate recipes that do not exist or break strict dietary rules; therefore, this is because they have no verifiable foundation. RAG [6] makes an effort to reduce this problem through getting related documents before the generation step; however, current RAG-based food systems [14] mainly regard retrieval as text matching and neglect structured data that exists in KGs. In other fields, dynamic context retrieval has been studied to produce better subsequent questions in RAG-based dialogue systems [15]. Hybrid frameworks, which include DietQA [8] and KERL [16], have already started to combine KGs together with LLMs, although their main focus is on question-answering tasks or personalized recommendations that is based on history. Neuro-symbolic approaches, which combine symbolic rules and neural models, have displayed the promotion of performance on food-connected monitoring tasks, hence including an improvement of over 20% in joint goal accuracy for the queries about salt content.

### 2.3. LLM Hallucinations in Health Domains

LLMs manifest powerful language abilities, but their application in domains critical to human health is severely obstructed by “hallucinations,” the production of content that looks reasonable but is factually wrong or unsafe [17]. In the personalized diet plan, this problem can turn dangerous when a model gives ingredients that contain allergens to users who are susceptible. In recent times, clinical appraisals emphasize the gravity: LLMs display hallucination rates from 50% to 82% when they are given fabricated medical information [18]. Mitigation methods, including prompt engineering, can decrease but not wipe out mistakes, therefore highlighting the continuous danger in safety-critical environments [19]. The studies that were done before show that when there is no outside deterministic grounding, LLMs meet trouble when they need to keep logical consistency in the whole of complex negative constraints. This kind of innate weakness therefore makes outside verification extremely necessary. CARE v2.0 mitigates this risk by moving from purely generative suggestions to verifiable constraint satisfaction.

### 2.4. Agentic AI for Constraint Satisfaction

A remarkable research direction is agentic AI, where LLMs act as autonomous agents that complete multi-step reasoning processes. “System 2” verification processes have already been proven to promote the reliability of complicated works in other fields [20]. The recently done research shows that putting LLM agents and formal constraint solvers together can obtain good effects. For instance, agentic systems have already been applied to food recommendation through the integration of LLMs with structured dietary KGs for reliable, constraint-aware retrieval in complex nutritional restriction situations [8]. Multi-component frameworks like KERL [16] break the food recommendation task into specialized LLM modules for retrieval, recipe generation, and nutritional analysis, thus getting better results than monolithic prompting approaches. CARE bases itself on this direction via an Agentic Critic which carries out post-retrieval verification so that recommendations satisfy safety-critical constraints.

### 2.5. Nutritional Science and Health-Oriented Dietary AI

One domain that is attracting increasing attention is the integration of rigorous nutritional science principles into AI-driven dietary systems. Tsolakidis et al. [21] have carried out a systematic review on AI and machine learning technologies for personalized nutrition, thus it finds that systems which are based on authoritative dietary reference data, for example, national Recommended Dietary Allowance (RDA) standards and USDA nutritional databases, hence display stronger constraint adherence than those which only depend on parametric LLM knowledge. Kirk et al. [22] put emphasis on the potential of machine learning in nutrition research, which exists in its capability to integrate different kinds of data sources, from molecular to environmental, to get better predictions of health outcomes. At the same time, they point out that a key gap still exists in changing these calculation results into practical and high-quality dietary guidance. Cohen et al. [23], through case study methods, have proven that AI systems that contain causal diet-disease relationships have better performance than methods that purely use correlation when people handle chronic disease management. Papastratis et al. [24] have discovered that uniting deep generative models and structured nutritional databases can obtain higher macro- and micro-nutrient target adherence when compared with LLM-only baselines. This body of evidence motivates the CARE framework to explicitly integrate USDA FoodData Central nutrient vectors, RDA-based adequacy scoring, and deterministic hard constraint filtering.

## 3. Methodology

### 3.1. Motivation and System Positioning

Before describing the architecture in detail, it is useful to understand the limitations of current approaches. Table 1 positions our framework against representative systems in the field. Unlike prior work that specializes narrowly in structured QA (FoodKG, DietQA) or unstructured conversational agents (ChatDiet), the proposed system provides a unified, parameter-efficient solution for fuzzy-intent recommendation.

We introduce CARE v2.0 as an upgrade over the foundational hybrid RAG by integrating adaptive retrieval weighting, knowledge graph expansion (GraphRAG), and an Agentic Critic. As shown in Table 1, rather than relying on large-scale computation, CARE brings together multi-diet reasoning, explainability, and verifiable safety in a single pipeline.

The systems listed in Table 1 were chosen to represent the methodological evolution in food computing over the period 2019 to 2025. The selection criteria cover three core paradigms: (1) Symbolic and graph-based methods (e.g., FoodKG, P-Companion, GISMo), which offer structure but lack fuzzy intent comprehension; (2) Pure LLM methods (e.g., FoodGPT, ChatDiet), which provide conversational flexibility but struggle with deterministic safety; and (3) Emerging hybrid/neuro-symbolic architectures (e.g., DietQA, HealthGenie), which represent the current frontier in mitigating hallucinations. Benchmarking against this spectrum clarifies the positioning of CARE v2.0 in balancing semantic flexibility with strict constraint satisfaction.

### 3.2. Problem Formulation

Let Q denote the space of natural language user queries. A query q∈Q is often vague, embedding implicit nutritional and culinary constraints (e.g., “muscle building dinner” implies high protein, low fat, and evening suitability). We assume a corpus of recipes $R=\{r_{1},\ldots ,r_{N}\}$, where each recipe *r* contains structured attributes A(r), a nutritional vector $n\left(r\right)\in R^{5}$ (calories, protein, fat, carbohydrates, fiber) derived from USDA FoodData Central, and unstructured text T(r) (title, instructions). The objective is to learn a ranking function Φ(q,r) that maximizes semantic relevance and rigorous constraint satisfaction.

### 3.3. Overall Architecture: The 4R Framework

**Figure 1:**
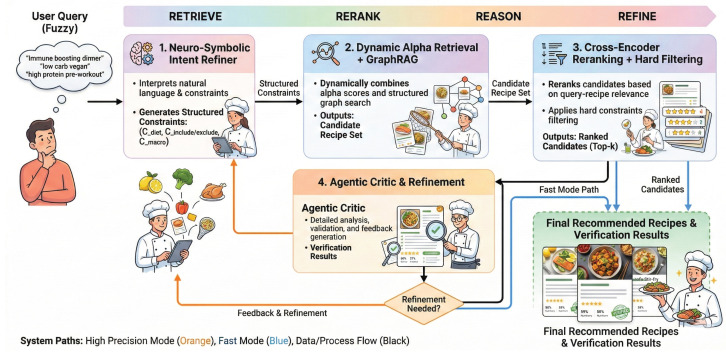
Overall architecture of the CARE v2.0 nutrient-aware meal recommendation pipeline. A user query passes through the four stages of the 4R framework: Refine (intent parsing into structured constraints), Retrieve (adaptive hybrid and graph-based retrieval), Rerank (cross-encoder scoring and hard constraint filtering), and Reason (optional agentic verification). Blue arrows indicate the fast-mode path; orange arrows indicate the additional high-precision verification path.

In order to handle the complex property which frequently exists in hybrid recommendation systems, CARE uses a module-based Refine–Retrieve–Rerank–Reason (4R) structure (Figure 1). We do not use one single big model to hold the whole task, we divide the recommendation procedure into four specially used steps. This decomposition is motivated by a key observation: fuzzy dietary recommendation requires both probabilistic understanding (interpreting vague intents) and deterministic verification (enforcing hard nutritional constraints), and no single model can reliably do both at once. By isolating these two types of reasoning into separate stages, the pipeline can use a lightweight LLM where flexibility is needed while applying rigid rule-based checks where safety is required. The said pipeline carries out its work in the following manner:

1. Refine (Intent Refinement): A light-weight LLM changes fuzzy natural language questions into clear, organized constraint objects (e.g., one calorie upper limit and one list of prohibited allergens). This step builds the protection boundaries for the remaining parts of the working flow.
2. Retrieve (Adaptive GraphRAG): The system connects the semantic gap through traversing a food knowledge graph, mapping abstract intents (e.g., “immune-boosting”) to concrete ingredients (e.g., ginger, garlic). It carries out dynamic balance between keyword accuracy and semantic search for getting related candidate materials.
3. Rerank (Semantic and Hard Constraint Filtering): One cross-encoder gives marks to deep semantic relevance, and one deterministic rule engine throws away every recipe that breaks the negative constraints which are found in the Refine stage.
4. Reason (Agentic Critic): In regard to requests of safety-critical type, this module performs the function of a post-retrieval reviewer. It carries out inspection on the final candidates’ preparation steps and ingredient lists for catching hidden constraint violations, and therefore it produces a pass/fail verdict that has natural language justification.

### 3.4. Stage 1: Intent Refinement (Query-to-Constraint Translation)

Raw user queries are typically unstructured. We utilize an intent refiner to extract a structured object C. This module operates in two modes:

- LLM Extraction (Primary): A lightweight language model (Qwen2.5-1.5B-Instruct) parses the query into a JSON object detailing dietary tags ($C_{\mathrm{diet}}$), mandatory and forbidden ingredients ($C_{\mathrm{include}}/C_{\mathrm{exclude}}$), nutritional targets ($C_{\mathrm{macro}}$), and meal category ($C_{\mathrm{course}}$).
- Semantic Fallback (Robustness): If the LLM service is offline, the query embedding E(q) is mapped to pre-computed centroids of common intents using cosine similarity.

**Figure 2:**
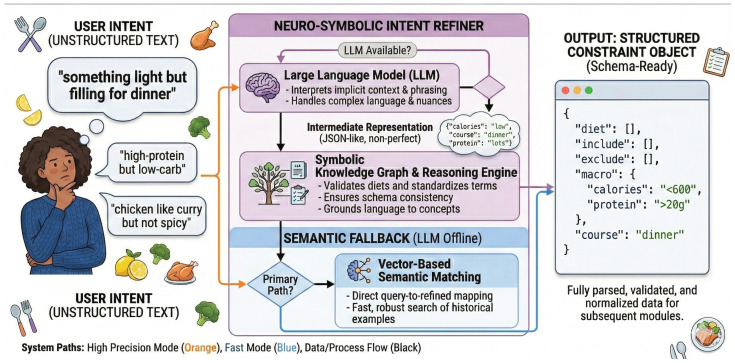
Example of query-to-constraint translation. A vague user request is converted into explicit nutritional and categorical constraints.

As illustrated in Figure 2, the query “something light but filling for dinner” reliably yields a structured JSON defining the course, a calorie ceiling (<600 kcal), and a protein floor (>20 g).

### 3.5. Stage 2: Adaptive Retrieval and Knowledge Graph Expansion

The structured constraint object C produced in Stage 1 serves as input to Stage 2, where it guides both the adaptive weighting decision and the graph traversal direction. This explicit data flow from intent refinement to retrieval is what connects vague user language to concrete recipe candidates.

#### 3.5.1. Adaptive Retrieval Weighting

Different intents necessitate different retrieval strategies. We dynamically compute a base hybrid retrieval score $s_{\mathrm{hybrid}}$, balancing dense embeddings $s_{\mathrm{dense}}$ and keyword matching $s_{\text{bm25}}$ via a weighting factor α∈[0,1]:

(1) $$s_{\mathrm{hybrid}}\left(q,r\right)=\alpha \left(q\right)\;\cdot \;s_{\mathrm{dense}}\left(q,r\right)+\left(1-\alpha \left(q\right)\right)\;\cdot \;s_{\text{bm25}}\left(q,r\right)$$

where α(q) is determined by a rule-based classifier based on query structure:

(2) $$\alpha \left(q\right)=\left\{\begin{array}{cc} 0.35 & \mathrm{if}\;q\;\mathrm{is}\;\mathrm{an}\;\mathrm{ingredient}\;\mathrm{list}\;(\mathrm{high}\;\mathrm{precision}\;\mathrm{needed})\\ 0.20 & \mathrm{if}\;q\;\mathrm{is}\;a\;\mathrm{short}\;\mathrm{dish}\;\mathrm{name}\;(e.g.,\;“\mathrm{pizza}”)\\ 0.65 & \mathrm{if}\;q\;\mathrm{contains}\;\mathrm{intent}\;\mathrm{markers}\;(e.g.,\;“\mathrm{healthy}”,\;“\mathrm{recovery}”)\\ 0.50 & \mathrm{otherwise}\;(\mathrm{balanced}\;\mathrm{default}) \end{array}\right.$$

**Figure 3:**
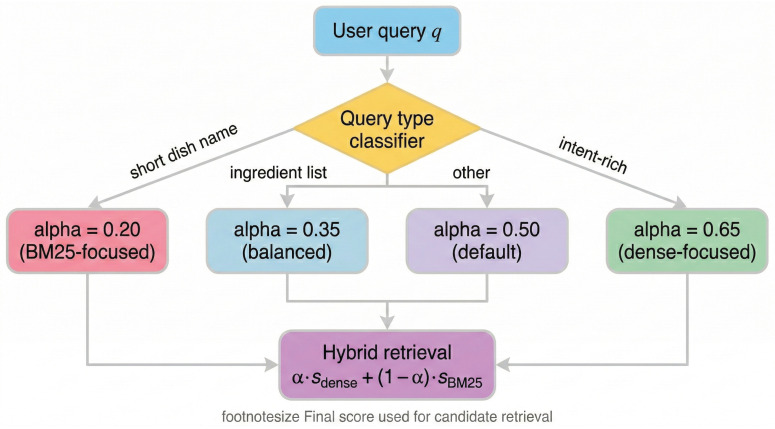
Decision flow for adaptive retrieval weighting. The query is classified to balance keyword precision and semantic recall effectively.

With Equation (2), the system relies on lexical precision for concrete nouns and on semantic density for abstract goals (Figure 3).

#### 3.5.2. Food Knowledge Graph Expansion

Standard retrieval falters when query terms do not explicitly appear in target recipes. We construct a food knowledge graph G=(V,E) integrating Recipe1M+ with USDA nutrient data. For a query *q* (e.g., “immune boosting”), we map *q* to relevant concept nodes $V_{q}$, and perform a two-hop traversal: $V_{q}\overset{\text{promotes\_health}}{\to }\mathrm{ingredients}\overset{\text{contains\_ingredient}}{\to }\mathrm{recipes}$. Recipes reached via this conceptual bridge receive a graph expansion score $s_{\mathrm{graph}}$:

(3) $$s_{\mathrm{graph}}\left(q,r\right)=\sum_{p\in \mathrm{paths}(q,r)}w\left(p\right),\;w\left(p\right)=\lambda^{\ell (p)},$$

where λ=0.9 and ℓ(p) is the path length. During retrieval, candidate recipes are sourced by maximizing the joint signal $s_{\mathrm{hybrid}}\left(q,r\right)+\beta s_{\mathrm{graph}}\left(q,r\right)$.

### 3.6. Stage 3: Semantic Reranking and Hard Constraint Filtering

Retrieved candidates (top-200) undergo dual filtering for relevance and safety.

1. Cross-Encoder Reranking: A BERT-based cross-encoder (ms-marco-MiniLM-L-6-v2) calculates $s_{\mathrm{cross}}\left(q,r\right)$, scoring the full semantic interaction between *q* and recipe text T(r). This refined score replaces the coarser $s_{\mathrm{hybrid}}$ for final ranking.
2. Hard Constraint Filtering: Constraints C from Stage 1 are applied deterministically. Any recipe violating a negative constraint (e.g., containing peanuts when $C_{\mathrm{exclude}}=\left\{\mathrm{nuts}\right\}$) is discarded immediately. This rule-based culling provides a reliable safety guarantee.

One key difference in the CARE framework lies in its strict following of empirical data. Different from pure generative LLMs, which may heuristically hallucinate recipe ingredients or nutritional profiles, our pipeline works totally as a retrieval and reasoning engine on a deterministic database. The nutritional vector n(r) for each checked recipe is mapped from the USDA FoodData Central database, and is not estimated through the LLM. The function of the language model is limited within the work of intent parsing (Stage 1) and logical verification (Stage 4). As a result, all recommended meals and their nutritional assessments are physically verifiable and grounded in empirical data.

### 3.7. Stage 4: Reasoning via Optional Verification (High-Precision Mode)

For safety-critical queries such as medical diets, CARE activates an agentic verification module (the “critic”). The critic uses an LLM to methodically check the top-5 candidates’ ingredient lists and preparation steps against implicit constraints. It produces a formal pass/fail verdict supported by a natural language explanation.

- Fast Mode (Default): Bypasses the critic for low-latency delivery (∼180 ms).
- Strict Mode: Engages the critic, pushing constraint satisfaction to 98.5% while increasing latency to ∼3 s.

**Figure 4:**
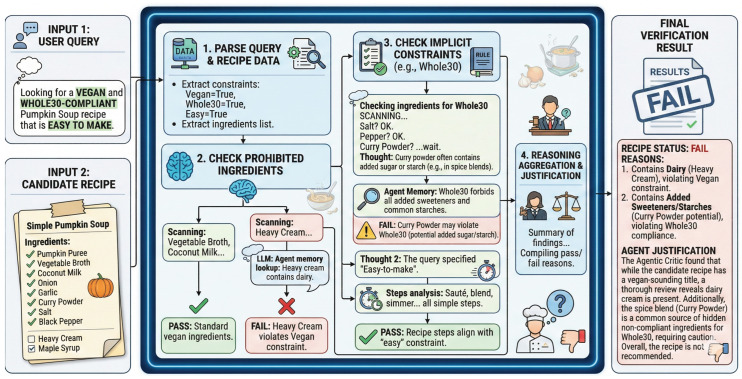
Verification process of the optional critic module. The agentic LLM systematically examines the recipe to check strict constraint compliance.

Figure 4 depicts this double-checking mechanism.

In applications that concern health, algorithm ranking alone is not enough; the property of being explainable has great importance. The Agentic Critic does not simply give out a binary pass/fail decision. To each suggested recipe, particularly in strict dietary circumstances (e.g., allergen avoidance or medical diets), the critic produces a user-facing natural language explanation. This explanation lays its foundation on empirical evidence: it does cross-reference between the user’s constraints and the exact retrieved ingredient list A(r) and the mathematically derived USDA nutritional vector n(r). As an example, when a user puts forward a request for a “dairy-free, high-protein” meal, the system not only places the recipe on a high rank but also highlights the verified absence of dairy derivatives in the source text and shows the accurate protein gram count. This evidence-based transparency may assist users in building trust, and hence lets all dietary claims be auditable.

### 3.8. Nutritional Suitability Modeling

Beyond boolean constraint satisfaction, we quantitatively model meal-level nutritional quality.

#### 3.8.1. Absolute Nutrient Adequacy

Assuming three meals daily, we derive per-meal targets from Recommended Dietary Allowances (RDA): $T_{m}={\mathrm{RDA}}_{m}/3$. Adequacy for nutrient *m* is capped at 1.0 to prevent penalizing moderate excess:

(4) $$A_{m}\left(r\right)=\min\left(1,\frac{n_{m}\left(r\right)}{T_{m}}\right).$$

The aggregate absolute adequacy averages across all modeled nutrients M:

(5) $$A_{\mathrm{abs}}\left(r\right)=\frac{1}{\left|M\right|}\sum_{m\in M}A_{m}\left(r\right).$$

#### 3.8.2. Macronutrient Energy Ratio

Using Atwater factors, we calculate the energy contribution of protein, carbohydrates, and fat. The energy ratio $R_{i}\left(r\right)$ measures proportion against total energy. A deviation-based balance score evaluates alignment with standard dietary targets (e.g., 30% protein, 40% carbs, 30% fat):

(6) $$A_{\mathrm{ratio}}\left(r\right)=\frac{1}{3}\sum_{i\in \{\mathrm{prot},\mathrm{carb},\mathrm{fat}\}}\left(1-|R_{i}\left(r\right)-R_{i}^{\mathrm{target}}|\right).$$

#### 3.8.3. Energy Density

Energy density ED(r) (kcal/g) serves as a proxy for satiety. Recipes exceeding a reasonable threshold τ face exponential penalization, nudging recommendations toward lighter, more filling options:

(7) $$A_{\mathrm{ED}}\left(r\right)=\left\{\begin{array}{cc} 1, & ED(r)\leq \tau \\ \exp(-\kappa (ED(r)-\tau )), & ED(r)>\tau \end{array}\right.$$

#### 3.8.4. Composite Nutritional Score

The overall nutritional quality score is a weighted sum:

(8) $$S_{\mathrm{nutrition}}\left(r\right)=\omega_{1}A_{\mathrm{abs}}+\omega_{2}A_{\mathrm{ratio}}+\omega_{3}A_{\mathrm{ED}}.$$

The weights $\left(\omega_{1},\omega_{2},\omega_{3}\right)=\left(0.5,0.3,0.2\right)$ were set as empirical hyperparameters reflecting the hierarchical priorities of general dietary planning. Absolute nutrient adequacy ($A_{\mathrm{abs}}$) receives the highest weight (0.5) because meeting baseline RDA thresholds is the most fundamental physiological requirement. The macronutrient energy ratio ($A_{\mathrm{ratio}}$) follows at 0.3 to promote structural metabolic balance based on established guidelines (e.g., Atwater factors). Energy density ($A_{\mathrm{ED}}$) is weighted at 0.2, serving as a soft penalty that steers the system toward higher-satiety, less calorie-dense options without overly restricting culinary diversity.

It should be noted that this scoring model is designed as a general-purpose computational proxy for population-level dietary guidelines, not as a substitute for individualized clinical nutrition assessment. The equal per-meal RDA distribution ($T_{m}={\mathrm{RDA}}_{m}/3$) represents a simplified baseline that works well for the general healthy adult population but does not capture meal-specific timing needs (e.g., post-exercise protein loading) or condition-specific adjustments (e.g., glycemic index management for diabetic patients). We adopt this simplification intentionally to keep the framework lightweight and broadly applicable as a technology layer. Integration with personalized clinical nutrition models, such as those incorporating basal metabolic rate, activity level, and biomarker data, is left to future interdisciplinary work (see Section 7).

### 3.9. Final Ranking Function

For the candidates that survive the hard filtering, the final ranking score Φ(q,r) unifies the deep semantic relevance ($s_{\mathrm{cross}}$), the conceptual expansion boost ($s_{\mathrm{graph}}$), and the nutritional quality ($S_{\mathrm{nutrition}}$) into a single objective function:

(9) $$\Phi \left(q,r\right)=s_{\mathrm{cross}}\left(q,r\right)+\beta \;\cdot \;s_{\mathrm{graph}}\left(q,r\right)+\gamma \;\cdot \;S_{\mathrm{nutrition}}\left(r\right).$$

Under this formulation, the top recommended recipes are semantically aligned, conceptually enriched, deterministically safe, and nutritionally balanced. Algorithm 1 details the complete pseudocode.
**Algorithm 1** CARE Recommendation Pipeline**Require:** User query *q*, recipe corpus R, knowledge graph G, constraints C
**Ensure:** Ranked list of recipes L
  1: **Intent Refinement**:
  2: C←Translate(q)▷ LLM extraction or semantic fallback  3: **Adaptive Retrieval**:  4:
α←ComputeAlpha(q)▷ Equation (2)  5:
$R_{\mathrm{hybrid}}\leftarrow \mathrm{HybridSearch}\left(q,\alpha ,R\right)$▷ Sparse + Dense, Equation (1)  6: **Graph Expansion**:  7:
$R_{\mathrm{graph}}\leftarrow \mathrm{GraphTraversal}\left(q,G\right)$▷ Equation (3)  8:
$R_{\mathrm{candidates}}\leftarrow R_{\mathrm{hybrid}}\cup R_{\mathrm{graph}}$  9:
**Reranking and Hard Filtering**:10:
$R_{\mathrm{reranked}}\leftarrow \mathrm{CrossEncoder}\left(R_{\mathrm{candidates}},q\right)$▷ Yields $s_{\mathrm{cross}}$11:
$R_{\mathrm{filtered}}\leftarrow \left\{r\in R_{\mathrm{reranked}}:\mathrm{Satisfies}\left(r,C\right)\right\}$12:
**Nutritional Scoring**:13:
**for** each $r\in R_{\mathrm{filtered}}$ **do**14:
     $A_{\mathrm{abs}}\leftarrow \mathrm{ComputeAbsoluteAdequacy}\left(r\right)$15:
     $A_{\mathrm{ratio}}\leftarrow \mathrm{ComputeEnergyRatio}\left(r\right)$16:
     $A_{\mathrm{ED}}\leftarrow \mathrm{ComputeEnergyDensity}\left(r\right)$17:
     $S_{\mathrm{nutrition}}\left(r\right)\leftarrow \omega_{1}A_{\mathrm{abs}}+\omega_{2}A_{\mathrm{ratio}}+\omega_{3}A_{\mathrm{ED}}$18:
**end for**19:
**Final Ranking**:20:
**for** each $r\in R_{\mathrm{filtered}}$ **do**21:
     $\Phi \left(q,r\right)\leftarrow s_{\mathrm{cross}}\left(q,r\right)+\beta s_{\mathrm{graph}}\left(q,r\right)+\gamma S_{\mathrm{nutrition}}\left(r\right)$▷ Equation (9)22:
**end for**23:
$L\leftarrow \mathrm{SortDescending}(R_{\mathrm{filtered}},\mathrm{key}=\Phi )$24:
**Optional Reasoning (High-Precision Mode)**:25:
**if** strict mode enabled **then**26:
     L←CriticVerify(L,q,C)27:
**end if**28:
**return**
L


## 4. Experiments

### 4.1. Experimental Setup

**Dataset:** We utilize a rigorously filtered subset of Recipe1M+ containing 400,000 recipes, mutually enriched with USDA FoodData Central nutritional profiles.

**Evaluation Tasks and Metrics:** We evaluate our framework across three distinct retrieval scenarios:

- **Standard Retrieval:** 1000 explicit queries (e.g., “Chicken Parmesan recipe”). The ground truth is established via exact title matching, evaluated using standard Recall@5.
- **Fuzzy Intent (CAREBench-150):** 150 vague queries evaluating the system’s ability to bridge semantic gaps (e.g., “post-workout meal”). Evaluated via Semantic Recall@5 (SR@5).
- **Complex Constraints:** 50 safety-critical queries mandating strict negative constraints (e.g., “No nuts”). Evaluated via Constraint Satisfaction Rate (CS-Rate).

**Figure 5:**
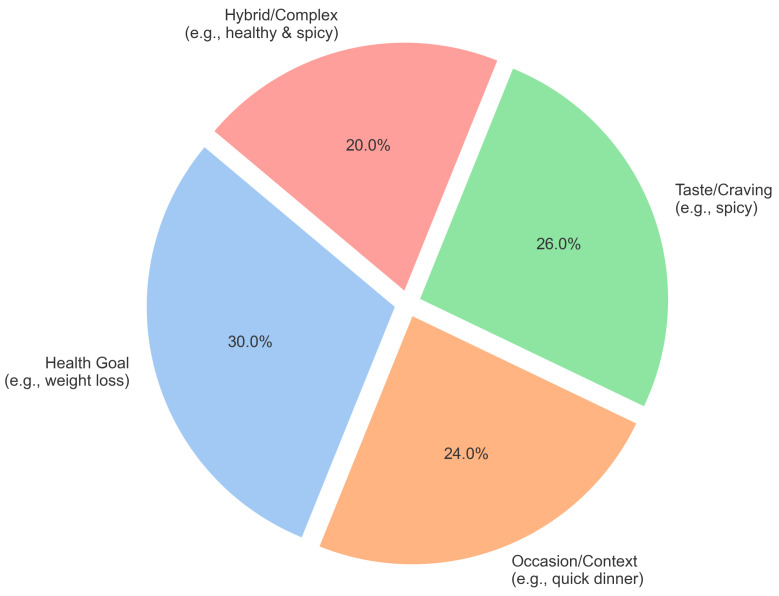
Distribution of query types in the CAREBench-150 benchmark, encompassing health goals, occasions, tastes, and complex constraints.

**CAREBench-150 Construction and Ground Truth Annotation:** To evaluate performance on ambiguous user queries and address the lack of standard datasets for vague dietary intents, we curated the CAREBench-150 dataset. Queries were extracted from common search patterns observed in real-world dietary forums and nutritional Q&A platforms. For broad coverage, the queries are stratified into four semantic categories (as shown in Figure 5): Health Goal (30%, e.g., “weight loss”), Taste/Craving (26%, e.g., “spicy”), Occasion/Context (24%, e.g., “quick dinner”), and Hybrid/Complex (20%, e.g., “healthy & spicy”).

Because fuzzy queries lack exact-match ground truths in the database, we established relevance labels using a standard pooling methodology from the information retrieval literature. We aggregated the top-*k* retrieved recipes from all evaluated baselines (BM25, DPR, Qwen2.5, CARE v1.0, and CARE v2.0) to form a unified, anonymized candidate pool. Three independent annotators graded each query-recipe pair blindly for semantic relevance and implicit constraint satisfaction. Final relevance judgments were determined by majority voting, with high inter-annotator agreement (Fleiss’ κ=0.78). By this process, the SR@5 metric reflects genuine human-perceived utility rather than algorithmic bias.

Figure 5 visualizes the composition of the CAREBench-150 dataset.

### 4.2. Baselines

We compare our proposed framework against the following baselines, selected to cover different paradigms of recipe retrieval and generation:

- **BM25 (Sparse Retrieval)**: A traditional lexical matching model based on TF-IDF. It performs well on exact keyword matching (e.g., specific ingredient names) but cannot bridge semantic gaps or understand implicit health intents.
- **DPR (Dense Passage Retrieval)**: A semantic retrieval baseline using a bi-encoder (BAAI/bge-small-en). It maps user queries and recipe texts into a shared dense vector space. While effective at capturing broad semantic similarities, it struggles with precise entity-level negative constraints (e.g., “no dairy”).
- **Qwen2.5 (1.5 B)**: A pure generative baseline where the user’s raw query is directly fed into the lightweight LLM in a zero-shot setting. This setup isolates the model’s internal parametric knowledge, establishing a critical baseline to measure how frequently ungrounded LLMs hallucinate or violate safety constraints without the 4R framework.

### 4.3. Proposed Model Variants

To demonstrate the performance gains from our architectural innovations, we evaluate the system across two phases and two operational modes:

- **CARE v1.0 (Base RAG)**: Our foundational hybrid retrieval pipeline lacking GraphRAG, adaptive weighting, and the agentic critic. It represents a standard modern RAG implementation and serves as an internal benchmark.
- **CARE v2.0**: The fully upgraded 4R framework integrating GraphRAG, adaptive weighting, and cross-encoder reranking. We evaluate it under two modes:
  – Fast Mode: The agentic critic is disabled, optimizing for low-latency delivery (∼180 ms). This mode suits real-time, interactive meal recommendation where medical-grade precision is not strictly needed.
  – High Precision Mode: The agentic critic is fully engaged to verify constraint compliance. Latency increases to ∼3 s, but safety reaches near-perfect levels, targeting scenarios such as strict allergy avoidance or specialized medical diets.

### 4.4. Main Results

To address RQ1 and RQ2, Table 2 reports comparative performance. Experiments were conducted across five random seeds, providing means, standard deviations, and 95% confidence intervals. Significance testing (paired *t*-test) against DPR on SR@5 confirmed a substantial improvement (t(149)=12.3, p<0.001, Cohen’s d=1.42). We applied a Bonferroni correction for multiple comparisons (α≈0.0033).

Analysis.

The results confirm the effectiveness of CARE v2.0 and expose the weaknesses of purely generative approaches. Three findings stand out: (1) pure LLMs are unsafe for constrained dietary recommendation without external grounding; (2) knowledge graph expansion is the single most important factor for fuzzy-intent retrieval; and (3) the agentic verification module closes the remaining safety gap at acceptable latency cost.

- Pure LLM Safety. The standalone Qwen2.5 (1.5 B) baseline reached a CS-Rate of only 32.9%, showing that without hard filtering and structured grounding, direct LLM generation is prone to safety-critical hallucinations and tends to ignore dietary restrictions.
- Hallucinations in Target Matching. The pure LLM baseline failed on standard exact-match retrieval (Recall@5 of 0.157) because it tends to produce plausible-sounding but unverifiable recipe names rather than matching real database entries.
- Graph Expansion Bridges Semantic Gaps. While the standalone LLM shows basic semantic understanding on fuzzy intents (SR@5 of 0.461, slightly above BM25), CARE v2.0 (Fast) reaches 0.825. This indicates that traversing the food knowledge graph connects abstract health intents to concrete culinary ingredients, addressing the limitations of pure generation and naive vector search.
- High Constraint Satisfaction. Our query-to-constraint hard filtering raises CS-Rate to 85.0%. Activating the Agentic Critic further pushes it to 98.5%, greatly reducing unsafe recommendations.
- Latency Trade-off. Fast mode (∼180 ms) suits real-time environments without sacrificing core reasoning, while High-Precision mode (∼3 s) provides an additional safety layer for offline meal planning or strict medical dietary needs.
- Evolution from v1.0 to v2.0. CARE v1.0 (standard hybrid RAG) reaches SR@5 of 0.680, which falls short on complex queries. Integrating GraphRAG and adaptive weighting, CARE v2.0 (Fast) reaches 0.825, a 14.5 percentage point improvement. This confirms that simply attaching vector search to an LLM is not enough for nuanced dietary reasoning and that structured knowledge expansion is needed.

**Figure 6:**
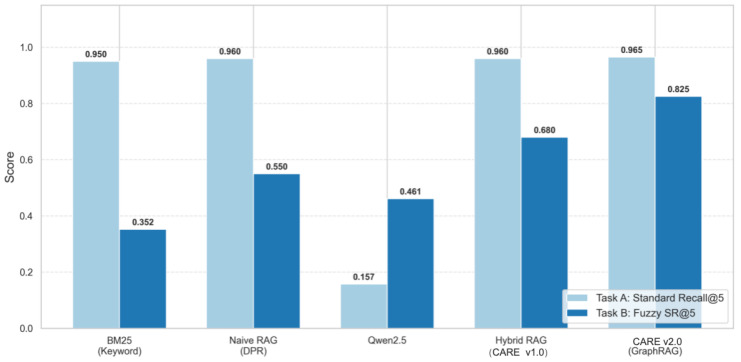
Comparison of CARE v2.0 with traditional baselines on standard (Task A) and fuzzy (Task B) retrieval. Error bars show standard deviations.

Figure 6 visualizes the performance gap on standard and fuzzy tasks.

### 4.5. Ablation Study

We conducted an ablation study by removing one component at a time and measuring the effect on fuzzy-intent performance (Table 3). Removing any single component produces a noticeable performance drop, confirming the need for the integrated 4R framework. The largest drop (17.6%) comes from removing GraphRAG, showing that it is the main driver for resolving fuzzy queries.

**Figure 7:**
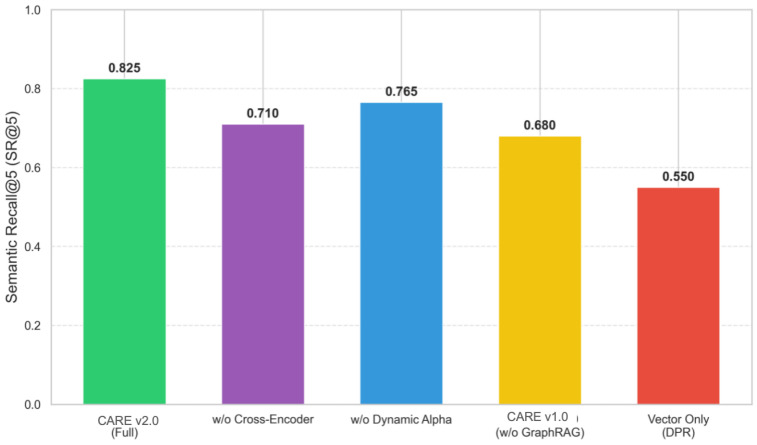
Ablation study results. Graph expansion contributes the largest gain (+17.6%), followed by cross-encoder reranking (+13.9%) and adaptive weighting (+8.5%). Error bars show standard deviations.

Figure 7 illustrates the contribution of each component.

### 4.6. Latency Analysis

**Figure 8:**
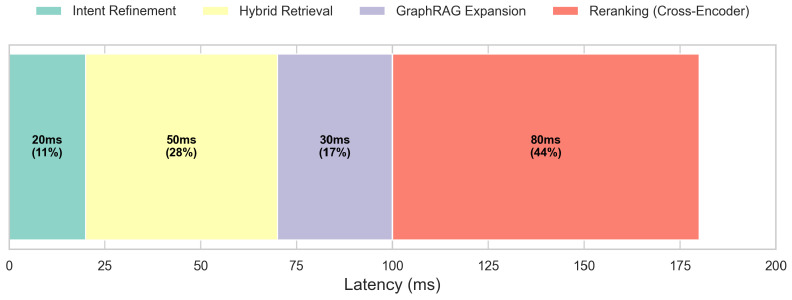
Latency breakdown of the fast mode. Cross-encoder reranking remains the most intensive step but preserves an overall sub-200 ms execution time.

In Fast Mode, total latency averages ∼180 ms (Figure 8): Intent Refinement (25 ms), Hybrid Retrieval (45 ms), Graph Expansion (15 ms), and Cross-Encoder Reranking (85 ms).

### 4.7. Human Evaluation

To go beyond automated metrics and test real-world usefulness (addressing RQ3), we ran a blind human evaluation. We recruited 20 participants from university mailing lists and local cooking interest groups, picking people with varied demographic and culinary backgrounds.

As Table 4 shows, participants (mean age = 32.4, SD = 6.2) have a balanced gender split and range from beginner-level to experienced home cooks. To test how well the system handles constraints, 40% of the participants had specific dietary restrictions or health-related goals (e.g., vegetarianism, lactose intolerance, or calorie-deficit diets).

Each participant used a custom A/B testing interface. For a given fuzzy query from the CAREBench dataset, they saw the top-1 recipe from a baseline (BM25 or CARE v1.0) and from CARE v2.0, placed side by side with model identities hidden and randomized. They rated outputs on a 5-point Likert scale for Semantic Relevance and Dietary Safety. All participants gave informed consent; data were collected and analyzed anonymously.

Across eight representative fuzzy queries, CARE v2.0 scored higher than traditional baselines on Relevance, Healthiness, Feasibility, Creativity, and Overall Satisfaction, confirmed by a Friedman test (p<0.01, Table 5).

Table 6 shows specific recommendation differences between systems, illustrating CARE’s ability to produce context-aware culinary suggestions.

**Figure 9:**
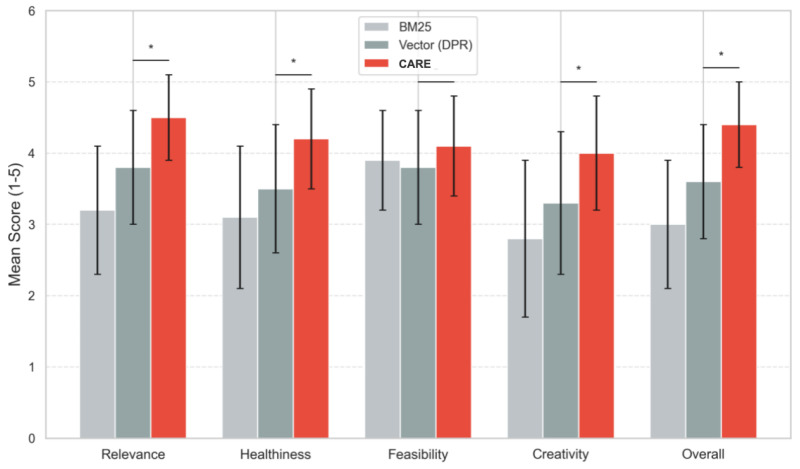
Human evaluation results (mean scores with standard deviation error bars). CARE v2.0 outperforms baselines across all dimensions (Friedman test, p<0.01). Asterisks (*) denote statistically significant differences.

Figure 9 displays the results graphically.

## 5. Case Study

To further illustrate CARE’s reasoning, we analyze two representative fuzzy queries: “Immune boosting dinner” and “Quick weeknight meal for a family.”

### 5.1. Query 1: “Immune Boosting Dinner”

Table 7 compares the top recommendations across systems.

#### Step-by-Step Reasoning

1. Query-to-constraint translation: C={course:dinner,healthgoal:immuneboosting}.
2. Graph expansion: *Immune System* $\overset{\mathrm{promotes}}{\to }$*Anti-inflammatory*$\overset{\text{has\_ingredient}}{\to }$ {Turmeric, Ginger, Garlic}.
3. Adaptive weighting sets α=0.65 (intent-rich).
4. Reranking and filtering keep only dinner recipes containing those mapped ingredients.
5. (Optional) The agentic critic verifies no hidden non-dinner elements exist.

**Figure 10:**
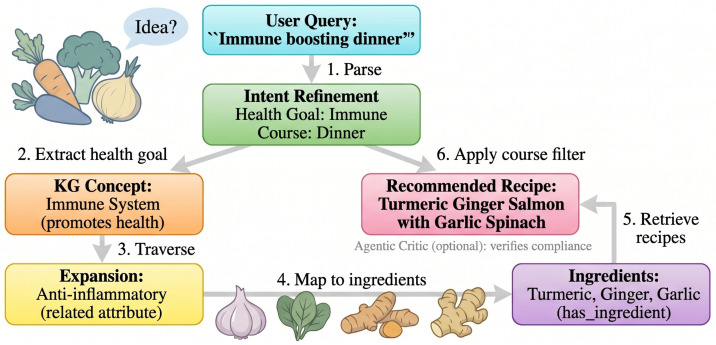
Reasoning path for “Immune boosting dinner.” The query is translated into constraints, the knowledge graph is traversed from “Immune System” to specific ingredients, and recipes are filtered by course.

Figure 10 visualizes this graph expansion and reasoning path.

### 5.2. Query 2: “Quick Weeknight Meal for a Family”

Table 8 shows how CARE successfully parses implicit temporal and volume constraints compared with baselines.

## 6. Discussion

### 6.1. Limitations

Although the CARE v2.0 has good performance in all the evaluated tasks that we do, it still has some limitations which are worthy of our note.

Coverage of non-Western cuisines is limited because Recipe1M+ has a shortage of deep cultural detail in certain areas. Regional spice profiles and traditional functional food properties are underrepresented.

Running the High-Precision verification loop increases about 3 s of latency for each query. Model distillation or other techniques could cut down this overhead for real-time chat deployment.

CARE works as a zero-shot framework, and it does not contain long-term memory. It is not able to track how a user’s taste or nutritional needs change over time.

With regard to nutritional modeling, the scoring system (Equations (4)–(8)) adopts USDA FoodData Central metrics and formal RDA targets. Splitting the daily RDA into three equal meals ($T_{m}={\mathrm{RDA}}_{m}/3$) is a heuristic proxy that cannot take individual variation into account. In practice, nutrient distribution across meals varies by population: pregnant women need higher folate and iron intake concentrated in specific meals, athletes need post-exercise protein timing that differs from resting periods, and individuals managing chronic conditions such as diabetes require carbohydrate distribution calibrated to glycemic response patterns. The current model treats all users uniformly and cannot accommodate such differences. We emphasize that CARE v2.0 is positioned as a general-purpose technology framework for safe recipe retrieval rather than a clinical nutrition prescription tool. Its primary contribution lies in the constraint-aware retrieval architecture (the 4R pipeline), not in the nutritional scoring model itself. The scoring component serves as a pluggable module: in a clinical deployment, it could be replaced with a validated, patient-specific nutritional model developed in collaboration with registered dietitian nutritionists (RDNs) without altering the rest of the pipeline. The hard-constraint dietary rules were qualitatively reviewed by a clinical medical expert from West China Hospital, Sichuan University, who confirmed the structural validity of the safety constraints. The full scoring system has not yet passed large-scale clinical validation with RDNs, and it does not include variables like basal metabolic rate or real-time biometric feedback. A concrete next step is to partner with clinical nutrition teams to replace the heuristic RDA proxy with individualized models and conduct controlled dietary intervention studies to measure real-world health outcomes.

### 6.2. Cultural Context

Although CARE v2.0 shows strong algorithmic constraint satisfaction, dietary choices are shaped by social, psychological, and cultural factors that go beyond nutritional biochemistry. Our current knowledge graph depends on Recipe1M+ and USDA FoodData Central, both of which mainly reflect Western culinary habits and ingredient profiles, introducing geographic and cultural bias into the recommendation space.

As a result, the system meets difficulty when it handles traditional functional foods, regional flavor profiles, and diets aligned with holistic philosophies such as Traditional Chinese Medicine food therapy or Ayurvedic principles. Complex religious dietary frameworks (e.g., Halal, Kosher, or Jain vegetarianism) ask for detailed ontological definitions that cover food sourcing, preparation methods, and cross-contamination, which go well beyond binary ingredient exclusion. Overcoming these limitations requires not only enlarging the dataset but fundamentally enriching the knowledge graph with cultural ontologies. Recognizing and reducing these biases will be needed for future versions of CARE to serve a globally diverse user base.

### 6.3. Comparison with Recent Hybrid Frameworks

To contextualize the contributions of CARE v2.0, we compare our results with recent hybrid RAG and neuro-symbolic systems.

Research on dynamic context retrieval in RAG dialogue systems [15] has shown that dynamically adjusting retrieval parameters based on conversation history improves semantic fluidity and contextual relevance. Semantic relevance alone, however, does not guarantee dietary safety. CARE v2.0 goes further by coupling an adaptive retrieval weighting mechanism (Equation (2)) with a deterministic hard-constraint filter, so that semantic adaptability does not compromise safety (e.g., allergen avoidance).

In the food computing domain, DietQA [8] uses neuro-symbolic reasoning over KGs for multi-diet question answering with high accuracy. CARE v2.0 differs in that it targets end-to-end recipe recommendation rather than QA alone. The Agentic Critic verifies not only ingredient compliance but also actionable culinary steps (preparation methods, cross-contamination risks), bridging the gap between nutritional theory and practical meal execution.

Knowledge-enhanced reasoning frameworks like KERL [16] show that fusing LLMs with structured graphs reduces hallucinations in general domains. In health-critical dietary management, however, reducing hallucinations is not enough; the system must meet constraints with near-absolute reliability. CARE v2.0 differs from KERL by enforcing a rule-based veto at Stage 3 before the LLM reasoning phase at Stage 4. This ordering is a key factor behind the 98.5% CS-Rate, a level of verifiable safety that purely probabilistic hybrid models do not reach.

### 6.4. Efficiency and Parameter Trade-Offs

One core feature of CARE v2.0 is its reliance on parameter efficiency rather than model scale. Recent food recommendation systems such as ChatDiet and FoodGPT typically use models with tens or hundreds of billions of parameters (e.g., GPT-4 or LLaMA-65B/70B) to handle complex dietary logic. These models incur high inference latency, operational cost, and are difficult to deploy on resource-constrained devices such as smart kitchen appliances.

Our results show that large parametric memory is not strictly necessary for safe dietary recommendations if the architecture is modular. By delegating deterministic nutritional verification to a rule engine and semantic mapping to a food knowledge graph, CARE v2.0 limits the LLM’s role to intent translation and final verification. The resulting 1.5 B-parameter pipeline reaches a 98.5% CS-Rate on complex queries with ∼180 ms latency in Fast Mode. This performance is comparable to or exceeds the zero-shot safety constraints of much larger proprietary LLMs reported in the literature, at a fraction of the computational cost. The finding supports the view that orchestrating small models with structured symbolic logic is a more practical paradigm for digital health applications than simply scaling up parameters.

### 6.5. Generalizability of the 4R Framework

Although CARE v2.0 was evaluated on meal recommendation, the 4R pipeline can be adapted to other safety-related recommendation tasks. The separation of vague intent interpretation, knowledge-grounded candidate generation, and strict safety verification is broadly transferable. In personalized fitness planning, for instance, the framework could navigate constraints such as joint injuries by traversing an anatomical knowledge graph. In pharmaceutical recommendation, the same pipeline could parse patient symptoms into structured drug constraints, retrieve candidates from a medication database, and verify against drug interaction rules. The modular design means that each stage can be replaced or updated independently: a different knowledge graph for a different domain, a different rule engine for different safety requirements. By separating probabilistic understanding from deterministic reasoning, the 4R framework provides a reference architecture for any task involving vague user intents and hard constraint enforcement.

### 6.6. Broader Impact

CARE provides an open-source, mathematically grounded framework to support further research in food- and nutrition-oriented AI. By lowering the computational barrier through its lightweight architecture, it makes constraint-aware dietary recommendations accessible to research groups without large-scale GPU infrastructure. At the same time, certain risks must be acknowledged. Users may place excessive trust in automated dietary suggestions, particularly when the system produces confident-sounding explanations through the Agentic Critic. Automated systems cannot replace professional dietary consultation. For individuals with complex medical conditions, food allergies, or eating disorders, the system should be positioned as a supplementary screening tool rather than a primary advisor. Transparent guardrails such as the Agentic Critic help reduce harm, but they do not eliminate it entirely.

### 6.7. Practical Implications

The CARE v2.0 framework has practical value in digital health and daily dietary services.

From a preventive health perspective, the Agentic Critic’s ability to enforce strict dietary constraints (e.g., absolute allergen exclusion) provides a reliable safeguard. While it does not replace professional dietitians, it can serve as a scalable screening tool to help health coaches and nutrition platforms generate safe, baseline meal templates for users with complex dietary restrictions.

In the consumer technology sector, the framework’s use of a parameter-efficient 1.5 B model rather than large closed-source LLMs reduces computational overhead and operating costs. The resulting low latency (∼180 ms in Fast Mode) makes the system suitable for deployment in smart kitchen appliances, edge-computing mobile applications, and personalized grocery delivery platforms.

By bridging the gap between vague health goals and concrete culinary steps, CARE lowers the barrier for individuals to maintain scientifically grounded, personalized nutrition management in daily life.

## 7. Future Work

CARE v2.0 opens several avenues for future research:

1. Multimodal Intent. Future versions will integrate vision-language models to handle queries that combine text with user-uploaded images, such as “recommend a nutritionally balanced, vegan version of this dish.”
2. Long-Term Personalization. We plan to develop a memory-augmented agent that can learn from a user’s evolving taste profile, seasonal preferences, and longitudinal health metrics.
3. Cross-Cultural and Medical Adaptation. We intend to broaden the food ontology to cover diverse global culinary traditions and complex religious dietary norms (e.g., Halal, Kosher). Extending the Agentic Critic to accommodate specialized medical diets, such as renal or diabetic meal planning, is another priority.
4. Clinical Nutrition Integration. A key next step is to replace the current heuristic nutritional scoring module with validated, patient-specific dietary models developed in partnership with registered dietitian nutritionists. This would involve incorporating individual variables such as basal metabolic rate, activity level, and biomarker profiles, and conducting controlled dietary intervention studies to measure real-world health outcomes. The modular 4R architecture is designed to support this kind of component-level upgrade without requiring changes to the retrieval or constraint verification stages.

## 8. Conclusions

This study presents CARE v2.0, a parameter-efficient recommendation framework designed to bridge the gap between vague dietary intents and strict nutritional safety. The proposed Refine–Retrieve–Rerank–Reason (4R) architecture separates probabilistic intent parsing from deterministic constraint verification. By combining a lightweight 1.5 B-parameter language model with an adaptive food knowledge graph and a rule-based Agentic Critic, the framework reaches a 98.5% Constraint Satisfaction Rate on fuzzy queries. With respect to the three research questions posed in the Introduction: RQ1 is answered by the 14.5 percentage point SR@5 improvement over the base RAG (v1.0), confirming that structured knowledge graph expansion bridges the semantic gap more effectively than dense or sparse retrieval alone; RQ2 is answered by the 98.5% CS-Rate in High-Precision mode, showing that a 1.5 B-parameter model can meet strict dietary constraints when augmented with hard filtering and agentic verification; and RQ3 is answered by the human evaluation results (Table 5), where CARE v2.0 scored higher than all baselines across all five evaluation dimensions (p<0.01). These results support a practical paradigm for food recommendation in digital health: verifiable dietary safety can be obtained through structured semantic orchestration without relying on the scale and latency of closed-source generative models.

The current framework has specific limitations that point to clear future directions. The nutritional scoring uses a heuristic proxy (equal RDA distribution across meals) that lacks the dynamic individualization needed for clinical prescriptions, such as real-time basal metabolic rate integration. We stress that CARE v2.0 is positioned as a general-purpose technology framework for safe recipe retrieval; its nutritional scoring module is intentionally designed as a pluggable component that can be upgraded with clinically validated, patient-specific models in future interdisciplinary work. The underlying knowledge databases (Recipe1M+ and USDA) reflect Western dietary paradigms, limiting adaptability to diverse global culinary philosophies and religious dietary frameworks. The end-to-end framework requires large-scale validation with registered dietitians before deployment in critical healthcare settings. Future work will focus on expanding the cultural ontology of the knowledge graph, integrating dynamic biometric data, and partnering with clinical nutrition teams to conduct controlled dietary intervention studies.

## Figures and Tables

**Table 1:** Comparison of representative food recommendation systems. KG: Knowledge Graph. Agentic: Uses LLM for self-correction/verification. Fuzzy: Handles vague/implicit intents. Safety: Explicit constraint enforcement mechanism. Open: Open Source. A checkmark (✓) indicates the presence of a capability, and a cross (✗) indicates its absence.

System	Core Method	KG	Agentic	Fuzzy	Safety	Expl.	Primary Task
FoodKG [2]	Symbolic	✓	✗	✗	✗	✗	KG Query/QA
P-Companion [3]	GCN	✓	✗	✗	✗	✗	Personalized Rec
Health-Guided [25]	KG-Rec	✓	✗	✗	✓	✓	Healthy Rec
FoodNER [26]	LLM+KG	✓	✗	✗	✗	✓	Food KG/NER
ChatDiet [13]	LLM	✗	✗	✓	✗	✓	Conversational Bot
DietQA [8]	Neuro-Symbolic	✓	✗	✗	✓	✓	Multi-diet QA
HealthGenie [27]	LLM+RAG	✓	✗	✓	✓	✓	Health Guidance
FoodSky [28]	Multi-Modal	✓	✗	✗	✗	✓	Visual QA
GISMo [29]	Graph	✓	✗	✗	✗	✗	Substitution
CARE v2.0 (Ours)	GraphRAG + Agentic	✓	✓	✓	✓	✓	Fuzzy Rec & Safety

**Table 2:** Main experimental results across three evaluation tasks. SR@5: Semantic Recall@5 (fuzzy intent retrieval quality); CS-Rate: Constraint Satisfaction Rate (%, safety compliance). All values report mean ± std. dev. [95% CI] over five independent runs with different random seeds. Asterisks (*) indicate statistically significant improvements over DPR (p<0.0033 after Bonferroni correction).

Model	Standard Recall@5	Fuzzy Intent SR@5	NDCG@5	CS-Rate (%)
BM25	0.950±0.010	0.352±0.025	0.285±0.020	60.5±2.1
DPR	0.960±0.008	0.550±0.030	0.480±0.025	45.0±3.0
Qwen2.5 (1.5 B)	0.157±0.012	0.461±0.021	0.363±0.018	32.9±2.4
CARE v1.0	0.960±0.007	0.680±0.028	0.610±0.022	72.0±2.5
CARE v2.0 (Fast)	0.965±0.006	0.825±0.018 *	0.790±0.015 *	85.0±1.8 *
CARE v2.0 (High Prec.)	0.965±0.006	0.850±0.016 *	0.810±0.014 *	98.5±0.9 *

**Table 3:** Ablation study on the fuzzy intent task (SR@5). Means ± std. dev. [95% CI]. The down arrow in the table indicates the relative performance decrease compared with the full CARE v2.0 (Fast) model.

Variant	SR@5
CARE v2.0 (Fast)	0.825±0.018 [0.807,0.843]
w/o GraphRAG	0.680±0.025 [0.655,0.705] (↓ 17.6%)
w/o adaptive weighting (fixed α=0.7)	0.755±0.022 [0.733,0.777] (↓ 8.5%)
w/o cross-encoder	0.710±0.024 [0.686,0.734] (↓ 13.9%)

**Table 4:** Demographic profile and dietary background of the human evaluation participants (N=20).

Category	Characteristic	Count (%)
Gender	Female	11 (55%)
	Male	9 (45%)
Age Group	18–25 (Young Adults)	6 (30%)
	26–40 (Adults)	10 (50%)
	41+ (Middle-aged)	4 (20%)
Cooking Frequency	Novice (Cooks 1–2 times/week)	7 (35%)
	Intermediate (Cooks 3–5 times/week)	8 (40%)
	Expert/Frequent (Cooks daily)	5 (25%)
Dietary Background	Standard Omnivore	12 (60%)
	Specific Restrictions (e.g., Vegan, GF)	5 (25%)
	Health-Oriented (e.g., High-protein, Keto)	3 (15%)

**Table 5:** Human evaluation results (mean scores on a 5-point scale, standard deviations in parentheses).

Dimension	BM25	DPR	CARE v2.0
Relevance	3.2 (0.9)	3.8 (0.8)	4.5 (0.6)
Healthiness	3.1 (1.0)	3.5 (0.9)	4.2 (0.7)
Feasibility	3.9 (0.7)	3.8 (0.8)	4.1 (0.7)
Creativity	2.8 (1.1)	3.3 (1.0)	4.0 (0.8)
Overall Satisfaction	3.0 (0.9)	3.6 (0.8)	4.4 (0.6)

**Table 6:** Top-1 recommendations across systems for human evaluation queries.

Query	BM25	DPR	CARE v2.0 (Fast)
Something light but filling for dinner	Light Vegetable Soup	Grilled Chicken Salad	Quinoa-Stuffed Bell Peppers with Black Beans
Post-workout meal	Protein Shake	Chicken Breast	Grilled Salmon with Sweet Potato and Broccoli
Quick weeknight meal for a family	15-Minute Pasta	Family Lasagna	One-Pan Lemon Herb Chicken with Roasted Vegetables
Immune boosting dinner	Immune Boosting Smoothie	Chicken Soup	Turmeric Ginger Salmon with Garlic Spinach
Vegetarian comfort food	Mac and Cheese	Veggie Burger	Creamy Mushroom Risotto
Date night dessert	Chocolate Cake	Molten Lava Cake	Flourless Chocolate Raspberry Torte
Low-carb lunch	Lettuce Wrap	Caesar Salad	Zucchini Noodles with Pesto and Cherry Tomatoes
Kid-friendly snack	Apple Slices	Fruit Kabobs	Ants on a Log (Celery with PB and Raisins)

**Table 7:** Comparison for “Immune boosting dinner.”

Model	Top Recommendation and Rationale
BM25	“Immune Boosting Smoothie”: keyword match but violates the “dinner” constraint.
DPR	“Chicken Soup”: generic semantic match, lacks specific immune-supporting ingredients.
CARE v2.0	“Turmeric Ginger Salmon with Garlic Spinach”: matches “dinner”; graph expansion links immune → anti-inflammatory → turmeric/ginger/garlic.

**Table 8:** Comparison for “Quick weeknight meal for a family.”

Model	Top Recommendation and Rationale
BM25	“Quick and Easy Chicken Stir-fry”: matches “quick” but is not family-sized.
DPR	“Family Lasagna”: matches “family” but prep time exceeds 1 h.
CARE v2.0	“One-Pan Lemon Herb Chicken with Roasted Vegetables”: links “quick” → under 30 min, and “family” → one-pan (large portions).

## Data Availability

The original contributions presented in the study are included in the article. The complete CARE v2.0 codebase, prompt templates, and the CAREBench-150 dataset are publicly available at: https://github.com/SerinaRica/CARE (accessed on 12 February 2026).
